# Precision Revisited: Targeting Microcephaly Kinases in Brain Tumors

**DOI:** 10.3390/ijms20092098

**Published:** 2019-04-28

**Authors:** Gianmarco Pallavicini, Gaia E. Berto, Ferdinando Di Cunto

**Affiliations:** 1 Neuroscience Institute Cavalieri Ottolenghi, 10126 Turin, Italy; gianmarco.pallavicini@unito.it (G.P.); gaia.berto@unito.it (G.E.B.); 2 Department of Neurosciences, University of Turin, 10126 Turin, Italy; 3 Department of Molecular Biotechnology and Health Sciences, University of Turin, 10126 Turin, Italy; 4 Neuroscience Institute of Turin (NIT), 10126 Turin, Italy

**Keywords:** microcephaly, kinase, brain tumor, glioblastoma multiforme, medulloblastoma

## Abstract

Glioblastoma multiforme and medulloblastoma are the most frequent high-grade brain tumors in adults and children, respectively. Standard therapies for these cancers are mainly based on surgical resection, radiotherapy, and chemotherapy. However, intrinsic or acquired resistance to treatment occurs almost invariably in the first case, and side effects are unacceptable in the second. Therefore, the development of new, effective drugs is a very important unmet medical need. A critical requirement for developing such agents is to identify druggable targets required for the proliferation or survival of tumor cells, but not of other cell types. Under this perspective, genes mutated in congenital microcephaly represent interesting candidates. Congenital microcephaly comprises a heterogeneous group of disorders in which brain volume is reduced, in the absence or presence of variable syndromic features. Genetic studies have clarified that most microcephaly genes encode ubiquitous proteins involved in mitosis and in maintenance of genomic stability, but the effects of their inactivation are particularly strong in neural progenitors. It is therefore conceivable that the inhibition of the function of these genes may specifically affect the proliferation and survival of brain tumor cells. Microcephaly genes encode for a few kinases, including CITK, PLK4, AKT3, DYRK1A, and TRIO. In this review, we summarize the evidence indicating that the inhibition of these molecules could exert beneficial effects on different aspects of brain cancer treatment.

## 1. Background

### 1.1. The Hurdles of High-Grade Brain Tumor Precision Medicine

High-grade brain tumors (HGBTs) are very aggressive cancers that represent an important unmet medical challenge. Medulloblastoma (MB) is the most common pediatric HGBT, and it also occurs in adults, although less frequently. Based on microarray and genomic sequencing technologies, MB has been classified into four biological subgroups (WNT, SHH, group 3, and group 4) [1,2]. MB is currently treated with surgery, followed by irradiation of the entire neuraxis and high-dose multiagent chemotherapy. The long-term survival rate can be as high as 90% in the rare WNT subgroup, but it is usually around 50% in most of the other cases, with the worst prognosis in group 3 and 4 patients [3,4]. Thus, many patients still die despite treatment, and those who survive suffer from neurological, cognitive, and endocrine disorders caused by the aggressive therapy [3,4].

In adulthood, the most frequent HGBTs are gliomas. Among them, glioblastoma multiforme (GBM) is one of the deadliest human cancers. According to the WHO, GBM accounts for approximately 12%–15% of all brain tumors, and 60%–70% of astrocytic tumors [5]. The standard therapy for GBM is mainly based on surgical resection in combination with radiotherapy and chemotherapy with alkylating agents, such as temozolomide (TMZ). Gene expression profiling has allowed for the classification of GBM into four distinct subtypes (i.e., proneural, neural, classical, and mesenchymal) associated with distinct genomic abnormalities and different responses to aggressive therapy [6]. Nevertheless, the longest median survival obtained in GBM patients treated with combined therapy has been 14 months [7].

For these reasons, more effective and specific therapies are urgently needed for HGBTs.

A common assumption is that the most straightforward strategy to develop new anticancer therapies is to directly target driver mutations, as well as molecular pathways connected to them. A paradigm for this approach is the dramatic improvement in therapy of chronic myeloid leukemia, determined by the introduction of ABL1 tyrosine kinase inhibitors [8], but the extension of this approach to other tumors faces many issues.

In the case of MB, targeted therapy has been developed for the SHH subtype. This subgroup, which represents approximately 30% of MB patients in children and more than 50% in adults, could take advantage of Vismodegib and other smoothened (SMO) inhibitors [9,10]. However, only a subgroup of these patients respond to treatment and, even in these cases, resistance rapidly develops [9,11]. As with many other pediatric cancers, MB is characterized by a low mutation burden [12], leading to a paucity of recurrent alterations. In addition, the recurrent mutations found in groups 3 and 4 involve *NMYC* amplification, *CTNNB1*, *PRDM6*, and *TERT* variants, which are difficult to target pharmacologically [13].

The current state of precision approaches is not better for GBM. In these tumors, many recurrent mutations are routinely identified, such as those involving growth factor receptors, MAPK, and PI3K/mTOR signaling pathways or inhibitors of cell cycle progression [6,14]. However, these variants have not been associated with clear prognostic and predictive results, challenging the assumption that they are strong cancer drivers. Even more disappointingly, no therapy against these targets has shown significant efficacy in clinical trials, probably as a consequence of cancer cell plasticity, redundancy among alterations, and intratumor genomic heterogeneity [15]. 

The application of immune checkpoint blockade strategies in HGBTs does not appear to provide much better perspectives. MB is not expected to be very responsive to these treatments, because of the low mutation burden and scarce inflammatory infiltrate [13]. On the other hand, despite encouraging preclinical results, clinical trials with PD1-PDL1 inhibitors have not shown a significant benefit in GBM, probably due to the strong immunosuppressive environment of these tumors [16].

A relatively unexplored alternative is to target molecules and mechanisms that, despite not being mutated, are nevertheless required for tumor growth, progression, and invasiveness [17]. Screening-based strategies have been proposed to identify cancer vulnerabilities in specific patients [18], but the time required for deploying such strategies is a strong barrier to their efficient practical implementation.

### 1.2. Congenital Microcephaly: A Tissue-Specific Phenotype of Ubiquitously Expressed Genes

A major problem for precision medicine is to understand whether and how the effects of tumor-driving mutations, as well as tumor responses to therapeutic agents, are rooted in the biology of cells that have undergone malignant transformation [19]. Specific epigenomic landscapes and local proteome composition may render a particular tissue or cell type permissive to particular oncogenic mutations, but may also result in tissue-specific vulnerabilities that could be exploited therapeutically [19]. 

On this ground, genes mutated in congenital microcephaly (CM) syndromes have been proposed as attractive targets for HGBT-directed drug development [20,21,22]. HGBTs originate from different types of neural progenitors. Although it is still debated from which precursors the different cancers originate, it is established that MB and GBM tumor cells share many molecular features with cerebellar granule progenitors and cortical radial glia cells, respectively [23,24]. The inactivation of genes associated with congenital microcephaly leads to specific alterations of proliferation and survival of such cells. 

CM is a heterogeneous group of disorders characterized by reduced head circumference at birth, to at least 3 standard deviations below the mean [25,26]. CM can be the result of nongenetic conditions, such as viral infections and toxic exposure, or it can be generated by rare genetic disorders [25]. Primary hereditary microcephaly (MCPH) is the simplest form of genetic CM, in which brain size reduction is accompanied by grossly normal brain architecture and mild to moderate intellectual disability [25,27]. The association of severe microcephaly and proportionate body growth reduction is instead characteristic of Seckel syndrome (SCKS). Pure MCPH and SCKS are rare conditions, since genetic CM is more often associated with syndromic features and comorbidities [25,26,28]. In the Online Mendelian Inheritance in Man (OMIM) database (https://www.omim.org), approximately 450 loci are linked to mendelian phenotypes in which microcephaly is a strong hallmark.

A striking common feature of these genes is that, during development, they are selectively required for proliferation and genomic stability of neural progenitors, despite being expressed in all proliferating cell types [29]. The biological basis of this specificity is only partially understood. In many cases, CM proteins are associated with centrosomes, and their loss leads to cell cycle and mitosis delay, mitotic failure, and randomization of spindle orientation [30]. These alterations may tilt the balance between symmetric and asymmetric divisions of neural stem cells, decreasing the pool of proliferating neural progenitors and/or increasing the frequency of premature commitment or terminal differentiation [30]. However, it has also been shown that the loss of many MCPH proteins leads to the accumulation of DNA damage and apoptosis [31,32,33]. Moreover, there is evidence that nongenetic insults associated with microcephaly could impinge on the same tissue-specific vulnerabilities [34]. Accordingly, oncolytic activity by the ZIKA virus has been proposed as a therapeutic strategy for GBM [35,36,37]. 

Regardless of the precise mechanisms, the inactivation of CM genes may specifically reduce the expansion of HGBT cells. As in normal neural progenitors, CM gene inhibition could impair cancer cell cycle progression, promote differentiation, and induce apoptosis, with marginal effects on normal cell types. Moreover, the inactivation of CM genes may sensitize HGBT cells to radiotherapy and chemotherapy [20]. 

Proof of concept about the suitability of MCPH genes as possible therapeutic targets has already been reported for *ASPM*, the gene mutated at the highest frequency in MCPH patients, as well as for *KIF14* and *CDK6*. ASPM loss has been found to arrest the proliferation of glioma stem cells [38], to radiosensitize GBM cell lines [39], and to reduce tumor growth in MB mouse models [32] by increasing DNA double strand break (DSB) accumulation [32,39]. Similar results have been obtained by inducing KIF14 depletion [40,41]. Finally, CDK4/6 inhibitors have displayed significant antineoplastic activity in pro-neural GBM cells in xenograft assays [42], and new inhibitors of these kinases capable of crossing the blood–brain barrier are actively being developed [43]. However, very little is known about the potential of other CM genes, as well as about the mechanisms that could influence their specificity.

**Figure 1:**
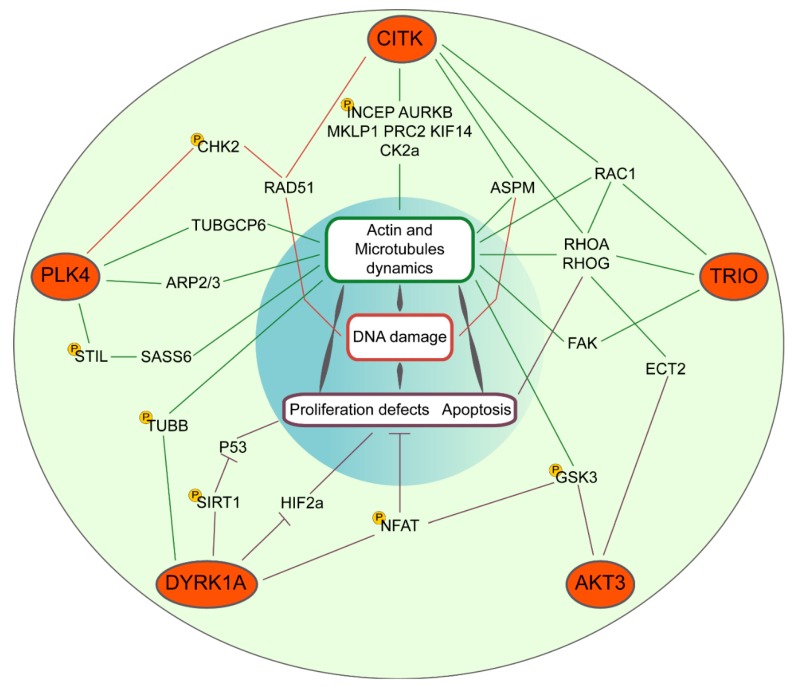
Convergent molecular pathways of microcephaly kinases CITK, PLK4, DYRK1A, AKT3, and TRIO. Their signaling cascades impinge, throughout the indicated common genes, on cytoskeletal dynamics and DNA damage responses or directly on proliferation and apoptosis. The indicated phosphorylation events (yellow P circles) have been experimentally proven.

CM-associated genes encode for some protein kinases. Considering their druggability, these proteins could represent a very interesting group of targets for HGBTs. Among them, CDK6 and CITK are involved in MCPH. On the other hand, mutations in ATR, PLK4, AKT3, DYRK1A, and TRIO have been associated with genetic disorders in which brain development is less specifically affected, leading to syndromic forms of CM. The potential of CDK6 and ATR as targets for MB and GBM has been deeply addressed in a recent review [22]. We therefore concentrate our survey on the remaining kinases (Figure 1). 

### 1.3. Citron Kinase (CITK)

CITK is a conserved AGC-type serine/threonine kinase. In mammals, it is the largest product of the *CIT* gene, with a molecular mass of 230 kD [44,45]. It displays a modular organization very similar to other members of the myotonic dystrophy kinase subfamily, comprising Rho-kinases (ROCKs) and CDC42BPA/CDC42BPB kinases (also known as MRCKs) [46]. These proteins share an amino-terminal kinase domain, followed by an extended coiled-coil region, a type 2 zinc finger, and a Pleckstrin homology domain (PH). CITK and MRCKs are characterized by a Citron-Nik1 homology (CNH) domain [45]. 

CITK is ubiquitously expressed in proliferating cells, with the highest levels in the G2/M phase of the cell cycle [47]. It is enriched at spindle poles before anaphase [48] and concentrates at cleavage furrows and midbody during cytokinesis [45]. The best studied function of CITK is to regulate midbody maturation and abscission at the end of cytokinesis [45,48,49,50,51], in concert with anillin (ANLN) [48], microtubule-binding proteins MKLP1, PRC2, and KIF14 (encoded by microcephaly gene *MCPH20*) [49], as well as chromosomal passenger complex (CPC) [52] and Casein kinase 2 [53]. CITK is also required for localization of F-actin at the abscission sites for the final cut of the midbody [51]. 

The only validated substrate of CITK is CPC component INCENP, whose phosphorylation by CITK regulates midbody organization by mediating a positive feedback loop between local CITK recruitment and AURKB activation [52]. CITK also regulates mitotic spindle orientation by interacting with ASPM (encoded by the microcephaly gene *MCPH5*) [48]. Finally, CITK prevents the accumulation of DNA double strand breaks independently of its role in cytokinesis and affects recruitment of RAD51 at DNA-damage foci [31]. 

Despite ubiquitous expression, CITK is functionally required in vivo only in a few cell types, including neural progenitors [54,55] and male germ cells [56]. Consequently, CITK loss leads to severe microcephaly in rodents [54,55] and humans [57,58,59,60]. Cells in the affected tissues display cytokinesis failure, apoptosis, and the accumulation of DNA damage [31,54,57,58]. The syndrome associated with CITK mutations is a particularly severe form of CM, known as *MCPH17.* Head circumference can be as low as 8 standard deviations below the mean, with moderate or severe intellectual disability. Many patients show a short stature and spasticity, and a few cases also display renal agenesis. Notably, in approximately half of *MCPH17* patients, homozygous missense mutations in the kinase domain have been found, resulting in a loss of catalytic activity [58]. Together with the other data, the latter evidence underscores that CITK activity is at the center of a complex interaction network essential for normal proliferation and survival of neural progenitors, comprising many other CM-associated proteins.

Concerning its possible role as a cancer drug target, CITK has long been a “neglected” protein [61], probably due to its highly specific developmental role. 

As with most microcephaly proteins, CITK is expressed at high levels in tumors [62,63,64], but this could likely be a reflection of cell-cycle regulated expression. Despite its strong tissue-specific requirement in normal cells, CITK knockdown negatively impacts the proliferation of tumor cell lines of different origins, in which it consistently induces cytokinesis failure, resulting in the accumulation of multinucleated cells [65,66]. 

The possible usefulness of CITK as a target for CNS tumor treatment has recently been explored in MB models [67]. CITK depletion by RNAi impairs in vitro expansion of MB cell lines and limits the growth of xenograft tumors. Moreover, temporally controlled deletion of CITK in tumors arising in the transgenic SmoA1 MB model reduces tumor growth and increases survival. In all models, CITK loss has been accompanied by cytokinesis failure, as well as by DNA damage and the induction of cell senescence. Interestingly, similar effects were obtained both in P53-proficient and P53-deficient cells [67]. At the moment, no data are available on the possible requirement for CITK in GBM cells, and no specific inhibitors of CITK have been reported.

### 1.4. Polo-Like Kinase 4 (PLK4)

PLK4 is one of the members of the Polo-like proteins, a kinase subfamily that plays a pivotal role in cell cycle progression and cytokinesis [68]. It is characterized by an N-terminal kinase domain, closely related to other PLKs. However, it shows a divergent carboxy-terminal domain, containing a single polo-box domain (PBD) instead of two tandem PBDs [68]. Polo boxes of PLK4 are involved in protein–protein interactions and control kinase activation, protein localization, and substrate specificity [69]. The PDB functions as a phosphoserine/threonine-binding module that has the highest affinity for Ser-[pSer/pThr]-[Pro/X], suggesting that PLK4 binds to docking sites primed by CDKs, MAP kinases, and other mitotic kinases [70]. 

In vivo expression of PLK4 is correlated with proliferation, and is very high in embryonic tissues and adult testes, moderate in the spleen and thymus, and not detectable in the brain, lungs, kidneys, breasts, heart, ovaries, and liver [71]. Expression peaks during the S, G2, and M phases of the cell cycle, while kinase activity is induced in the S phase and doubles from S to G2 [72]. 

PLK4 is localized in the nucleolus during G2, becomes enriched at centrosomes in the M phase, and concentrates at the midbody in cytokinesis [73]. In the centrosome, PLK4 is specifically concentrated at the proximal ends of the centriole outer wall and has also been observed close to the distal appendages of the mother centriole [74]. 

Consistent with its localization, PLK4 plays a fundamental role in centriole duplication [75]. PLK4 knockdown leads to centriole loss [75], while PLK4 overexpression increases centrioles’ number [74], producing abnormal spindle and mitotic abnormalities in both cases [76,77]. In mammals, centriole biogenesis is initiated by CEP192 and CEP152 (*MCPH9*), which recruit PLK4 to the proximal end of the mother centriole [78,79]. An interaction between PLK4 and STIL (*MCPH7*) results in STIL phosphorylation [80] and subsequent recruitment of SASS6 (*MCPH14*), which initiates nine-fold symmetric cartwheel nucleation and γ-tubulin assembly [81]. PLK4 also phosphorylates CHK2, a key transducer of ATM and ATR in the DNA damage response [82]. Therefore, PLK4 physically and functionally interacts with the products of many CM genes. 

Homozygous deletion of PLK4 in mice is embryonically lethal at the postgastrulation stage, with a marked increase in mitotic and apoptotic cells [73]. PLK4 +/− mouse embryonic fibroblasts have demonstrated a high rate of primary cytokinesis failure, associated with aberrant acto-myosin ring formation, reduced RHOA activation, and failure to localize the RHOA guanine nucleotide exchange factor ECT2 to the midbody [83]. 

Although these data suggest a direct involvement of PLK4 in cytokinesis, further studies have excluded this possibility, indicating that cytokinesis failure from decreased PLK4 function is only an indirect consequence of the spindle abnormalities caused by centrioles [84]. 

Despite PLK4′s house-keeping functions in mitosis, the identification of PLK4 mutations in microcephaly patients has provided evidence that neural progenitors are particularly sensitive to its levels. Homozygous truncating mutations in *PLK4* have been identified in seven affected members of a consanguineous family with autosomal recessive microcephaly, short statures, and chorioretinopathy, as well as in another two unrelated families [85]. A similar phenotype resulted from mutations in the PLK4 substrate TUBGCP6 [85]. Patient fibroblasts showed reduced centriole numbers, abnormal spindle formation, and decreased numbers of ciliated cells, correlating with the absence of basal bodies. However, patients did not show a ciliopathy phenotype [85]. In addition, patients’ cells showed genomic instability and altered DNA damage response [86].

There is abundant evidence that the alteration of PLK4 levels and activity may play a general driver role in cancer and that PLK4 expression is deregulated in many cancer types [69]. Notably, PLK4 acts as a tumor suppressor in haplo-insufficiency conditions by causing mitotic infidelity [87] and as an oncogene in overexpression conditions [88]. Centrosome amplification associated with PLK4 overexpression and its correlation with poor prognosis has been reported in many cancer types [69]. Moreover, the promotion of actin nucleation and invasiveness through phosphorylation of the Arp2/3 complex may contribute to PLK4 pro-metastatic potential [89]. 

These findings have led to rising interest in PLK4 as a promising and feasible target for cancer therapy and to the consequent development of PLK4 inhibitors. The first of such compounds was CFI-400945, which is capable of inducing mitotic defects and cell death in epithelial tumor cells [90]. In particular, CFI-400945 was reported to inhibit the growth of patient-derived pancreatic xenografts [91] and to induce polyploidy and cell death in lung cancer cells [92]. However, controversy exists about the specificity of this compound, because it leads to centrosome amplification, rather than the centrosome loss that would be expected from PLK4 inhibition [93]. This phenotype could be explained in part by the fact that CFI-400945 is also able to inhibit AURKB, leading to cytokinesis inhibition, in part by a feedback loop derived from partial PLK4 inhibition, preventing the degradation of autophosphorylated protein [94]. The latter mechanism could promote a paradoxical increase in PLK4 levels, with actual PLK4-dependent centrosome amplification [93]. 

A more specific compound is Centrinone, whose administration leads to total but reversible centrosome loss [95]. This compound has allowed for demonstrating that centrosomes are essential to the proliferation of normal cells, which undergo a permanent P53-dependent growth arrest upon Centrinone treatment [96]. In contrast, cancer cells continue to proliferate after centrosome loss, suggesting that centrosome depletion must be combined with other perturbations to selectively target them [93]. 

PLK4 is upregulated in embryonal CNS cancers, such as brain rhabdoid tumors [97], medulloblastoma [97,98], and neuroblastoma [99]. Rhabdoid cells within which PLK4 was targeted by CRISPR/CAS9 demonstrated significantly decreased proliferation, viability, and survival [100]. The PLK4 inhibitor CFI-400945 showed cytotoxic effects on rhabdoid tumor cell lines, while sparing non-neoplastic human fibroblasts and developing zebrafish larvae [100]. 

PLK4 inhibition has induced apoptosis, senescence, and polyploidy in MB cells, thereby increasing the susceptibility of cancer cells to DNA-damaging agents [97]. In malignant gliomas, elevated PLK4 levels were associated with poor prognosis and enhanced radio-resistance, while PLK4 knockdown significantly increased the radio-sensitivity of GBM cells [101]. The sensitivity of GBM cells to TMZ was also decreased by ectopic expression of PLK4 and was enhanced by PLK4 depletion and CFI-400945 treatment [102]. No reports are available on the effects of Centrinone in HGBT cells.

### 1.5. AKT Serine/Threonine Kinase 3 (AKT3)

AKT3 is one of three closely related serine/threonine-protein kinases, also called PKB, belonging to the family of AGC ser/thr protein kinases. These proteins share a conserved structure that includes three functional domains: an *N*-terminal PH domain, a central kinase domain, and a *C*-terminal regulatory domain containing the hydrophobic motif phosphorylation site [FxxF(S/T)Y] [103,104]. AKT1 and AKT2 play partially redundant roles in many processes of normal and cancer cells, including metabolism, proliferation, cell survival, growth, and angiogenesis [103,105,106]. 

AKT3 mRNA is expressed in many tissues, with the highest levels in the brain, testes, lungs, heart, kidneys, mammary glands, and fat [107]. It is activated by insulin through a PI3K-dependent mechanism requiring the PH domain and thr-305 phosphorylation [107,108]. AKT3 is the most represented AKT paralog in the brain during neurogenesis, and levels of phosphorylated pan-AKT are abundant in cortical progenitor cells during cortical development [109]. 

*Akt3* -/- mice show a selective 20% decrease in brain volume and hypoplasia of the corpus callosum, resulting from the reduction of both cell size and cell numbers [110,111]. This phenotype differs from *Akt1* -/- mice, in which brain size is reduced in the context of global body size decrease [110]. 

In consideration of the mouse knockout phenotype and its chromosomal localization (1q23-24), *AKT3* is considered the strongest candidate gene for 1q22-24 deletion syndrome, characterized by microcephaly and corpus callosum agenesis [112]. Identification of a balanced reciprocal t(1;13)(q44;q32) translocation in a patient with a similar phenotype, with a breakpoint close to the *AKT3* promoter, further supported this association [112]. Conversely, activating germline and somatic AKT3 mutations have been identified as a rare cause of megalencephaly and hemimegalencephaly, respectively [109,113,114]. 

The brain-specific role of AKT3 is at least partially explained by its expression pattern, since this kinase shares the same activation mechanisms and most substrates with its paralogs [115]. Thus, microcephaly could be explained by pro-proliferative and antiapoptotic effects on neural progenitors. However, it is possible that subtler mechanisms exist [115].

The AKT pathway is hyperactive in a large fraction of human cancers, including brain tumors [116]. Selective activation of AKT3 through overexpression or copy number increase is a recurrent event in nonfamilial melanomas [117]. AKT3 amplification has also been found in GBM [118] and MB [119]. AKT3 is required in transformed astrocytes and human glioma cells for anchorage-independent growth, and its loss has inhibited transformed cell invasion [120,121]. AKT3-expressing human GBM cells have shown enhanced activation of DNA repair proteins, leading to increased DNA repair and subsequent resistance to radiation and TMZ [122]. Accordingly, AKT3 knockdown had synergistic effects with TMZ and BCNU [123]. The development of therapeutic strategies based on selective and nonselective inhibitors of AKT3 and other AKTs represents an area of intensive investigation [116].

### 1.6. Dual Specificity Tyrosine Phosphorylation Regulated Kinase 1A (DYRK1A)

DYRK1A is a dual-specificity kinase regulated by tyrosine phosphorylation, belonging to the CMGC protein kinase family [124,125]. Human DYRK1A is a multidomain protein containing a highly conserved kinase domain preceded by a DH domain, which is characteristic of the DYRK kinase subfamily. In addition, it also contains two different nuclear localization signals, a PEST region, a histidine-rich region, and an extreme *C*-terminal region rich in serine and threonine. DYRK1A is capable of intermolecular activating autophosphorylation on tyrosine residues and phosphorylates its substrates on serine/threonine [124,126]. 

DYRK proteins are homologous to the *Drosophila minibrain* gene, and DYRK1A is widely known for its role in Down syndrome (DS). It is one of the more actively studied genes of the Down critical region, a relatively small part of human Chromosome 21 that plays a paramount role in DS-associated intellectual disability [125,127]. 

DYRK1A is expressed in most tissues, but during embryogenesis it is most abundant in the brain, spinal cord, and retina [128]. Homozygous deletion of *Dyrk1a* in mice is embryonically lethal, leading to general growth delay and death during midgestation. Heterozygous mice have shown decreased neonatal viability, reduced body size from birth to adulthood, and region-specific brain size decrease [129]. 

Accordingly, heterozygous *DYRK1A* deletion [130], as well as truncating and missense variants [131], have been found in patients showing microcephaly, intellectual disability, and autism spectrum symptoms. Both increased and decreased DYRK1A dosages have profound effects on neural progenitor biology. DYRK1A loss of function severely affects neural lineage specification [132], while the 1.5–2-fold increased expression that characterizes DS slows cell cycle progression, reduces progenitor pools, and impairs neuroblast differentiation in the developing neocortex [133]. 

Through the identification of substrates and interactors, DYRK1A has been involved in a broad range of cellular processes, including cell cycle regulation, cellular signaling, gene expression, chromatin modulation, alternative splicing, and membrane trafficking [134]. In particular, it has been shown to promote cell survival through different mechanisms, including inhibitory phosphorylation of Caspase 9 [135,136], priming phosphorylation of NFAT in the GSK3 pathway [137,138], and SIRT1 phosphorylation in response to genotoxic stress, in turn inhibiting TP53 activity and apoptosis [139]. Moreover, it may affect microtubule dynamics by phosphorylating β-Tubulin [140] and Tau [141].

Both tumor-suppressive and pro-oncogenic roles have been suggested for DYRK1A [134], especially in relation to brain tumors. In gliomas, it has been reported that DYRK1A may destabilize HIF2-alpha in hypoxic conditions by phosphorylating thr27 of ID2, leading to reduced self-renewal of glioma stem cells, the inhibition of tumor growth, and more favorable outcomes for patients with glioblastoma [142]. On the other hand, DYRK1A inhibition promotes EGFR degradation in primary GBM cells, thus compromising their survival and producing a profound decrease in tumor burden [143]. Accordingly, some novel, potent inhibitors (IC50 ≤50 nM) are capable of significantly decreasing viability, clonogenic survival, migration, and invasion of glioblastoma cells [144]. DYRK1A has also been shown to interfere with Shh/Gli signaling in MB [145].

### 1.7. Trio Rho Guanine Nucleotide Exchange Factor (TRIO)

The TRIO name derives from its sequence containing three main functional domains: two guanine nucleotide exchange domains for Rho-family small GTPases (GEFD1 and GEFD2), composed of a Dbl homology region followed by a PH domain, as well as one C-terminal ser/thr kinase domain [146]. In addition, TRIO comprises other modular units belonging to different structural classes [147]. TRIO was originally identified as an interactor of receptor tyrosine phosphatase LAR and is ubiquitously expressed with many different isoforms [146]. 

The principal functional regions of TRIO are the two GEFDs, which are capable of inducing actin remodeling through Rho GTPases. In particular, GEFD1 activates both RAC1 and RHOG, while GEFD2 acts specifically on RHOA [146,148]. The kinase domain is constitutively tyrosine-phosphorylated and interacts with LAR and with FAK. Increased tyrosine phosphorylation after FAK cotransfection increases TRIO association with the cytoskeleton [149]. 

A complete loss of function of TRIO is embryonically lethal in mice between E15.5 and birth, with a few escapers surviving for no more than one month [150]. *Trio* -/- mice display abnormal myotube fusion and aberrant organization in several brain regions, including in hippocampal formation and in the olfactory bulb [150]. 

Exome sequencing has revealed that *TRIO* is a haplo-insufficient gene, since heterozygous germline or de novo mutations have been found in several patients, characterized by delays in the acquisition of motor and language skills, mild to borderline intellectual disability, neurobehavioral problems, and microcephaly [151,152]. TRIO mediates axon outgrowth and guidance in response to extracellular cues transduced by different receptors. For instance, it may act downstream of the NGF receptor by activating RHOG through GEFD1 [153]. Moreover, it promotes signal transduction through axon-guidance receptor DCC, by favoring its membrane insertion and by becoming phosphorylated by tyrosine kinase FYN when DCC is bound by its ligand netrin-1. FYN-mediated tyrosine phosphorylation enhances the activity of GEFD1 toward RAC1, thereby promoting actin dynamics at the growth cone [154]. 

A very important function of TRIO for its involvement in cancer is to control cell adhesion and migration, especially downstream of Cadherin proteins [147]. For instance, both GEFD1 and GEFD2 are essential to mediate the collective migration of neural crest cells downstream of Cadherin-11. 

Upregulation of TRIO expression, often associated with poor patient survival, is found in different tumor types, including urinary bladder, breast, lung soft tissue sarcoma, and glioblastoma [147]. TRIO mediates glioma cell migration and invasion produced by stimulation of the TWEAK-Fn14 signaling axis by inducing RAC1 activation [155]. Accordingly, TRIO-directed siRNA oligonucleotides suppress glioblastoma cell migration and invasion and also reduce the rate of cell proliferation [156]. Compared to other kinases, TRIO is a more challenging target for pharmacological development, because the kinase domain is not crucial for function. Nevertheless, peptide aptamer-based [157] and small-molecule inhibitors [158], both interfering with the binding of GEF domains to cognate GTPases, are being developed.

## 2. Remarks and Conclusions

Sufficient evidence exists to support the notion that protein kinases associated with CM are promising targets for HGBT treatment. The results obtained through the inactivation or depletion of these proteins consistently have shown that, by interfering with microcephaly-related mechanisms, it is possible to decrease tumor cell clonal expansion, increase their sensitivity to chemotherapy and radiotherapy, and decrease their invasiveness. In many cases, experiments performed with the available inhibitors in vitro or with heterotopic xenograft models have provided proof of concept about their possible usefulness in therapy. However, many problems remain to be solved. In most of the studied cases, important pharmacological issues exist, especially concerning inhibitor development and/or delivery through the blood–brain barrier. Most inhibitors have not been tested yet in transgenic or orthotopic models, which more closely resemble the in vivo human condition. Since it is difficult to imagine that inhibitors of CM genes could be used as a monotherapy, a big effort is needed to address the effects of combining them with radiotherapy and chemotherapy, both in xenograft and orthotopic models. Moreover, it would be very interesting to study the effects of simultaneously inhibiting CM genes impinging on similar or different mechanisms. On these bases, although the inhibition of ubiquitous kinases may appear to be an old-fashioned approach, we are convinced that the underlying biological complexity may still offer a field with great potential.
